# Partial and Total Descemet's Detachments in a Patient with Severe Terrien's Marginal Degeneration and Juvenile Idiopathic Arthritis

**DOI:** 10.1155/2014/279491

**Published:** 2014-07-20

**Authors:** Amir Hossein Vejdani, Hamid Khakshoor, Michael V. McCaughey, Majid Moshirfar

**Affiliations:** ^1^Eye Research Center of Khatam Al-Anbia Hospital, Mashhad University of Medical Sciences, Mashhad 91959, Iran; ^2^University of New Mexico School of Medicine, Albuquerque, NM 87131, USA; ^3^John A. Moran Eye Center, 65 Mario Capecchi Drive, Salt Lake City, UT 84132, USA

## Abstract

A 16-year-old female with juvenile idiopathic arthritis presented with a one-month history of decreasing vision and increasing corneal edema in her left eye. Slit-lamp examination, keratometric measurements, and OCT evaluation led to a diagnosis of Terrien's marginal degeneration in both eyes along with a complete detachment of Descemet's membrane in the left eye and partial detachment in the right eye. She was treated with an intracameral injection of air and then topical betamethasone and chloramphenicol which lead to the resolution of symptoms. We further examine the pathophysiology of this disease based on current literature.

## 1. Introduction

Terrien's marginal degeneration (TMD) has traditionally been thought of as a rare, non-inflammatory, and slowly progressive disease. It is characterized by peripheral corneal thinning with intact epithelium, superficial vascularization, and lipid deposition. TMD typically occurs in males 40 years and older [1, 2]. Patients normally report an absence of eye pain, photophobia, or tearing [1]. However, a painful variant presenting in younger individuals has also been described [3]. In this case report, we present, to the best of our knowledge, the first reported case of Terrien's marginal degeneration associated with either juvenile idiopathic arthritis or Descemet's membrane detachment. As a separate entity, juvenile idiopathic arthritis has been associated with a miscellany of ocular complications such as macular edema, epiretinal membrane formation, hypotony, elevated intraocular pressure, band keratopathy, cataract formation, and uveitis with subsequent development of posterior synechiae [4].

## 2. Case History

A 16-year-old female presented to clinic with complaint of gradual vision loss over the previous month. She denied any ocular pain, photophobia, itching, or tearing. She had no history of contact lens use or family history of eye disease. Initial examination showed uncorrected visual acuity in her right eye to be 20/40 and counting fingers at ten centimeters in her left eye with no improvement in either eye on attempted correction. Extraocular movements were normal except for a sensory exotropia in the left eye. Past medical history was significant for polyarticular rheumatoid factor positive, ANA negative, and juvenile idiopathic arthritis (JIA) diagnosed at the age of five years. The disease initially affected her knees, wrists, and the metacarpophalangeal joints of her hands. In addition, an HLA B-27 analysis was negative. She was eventually placed on methotrexate and daily prednisone with relative success in the treatment of her symptoms.

**Figure 1:**
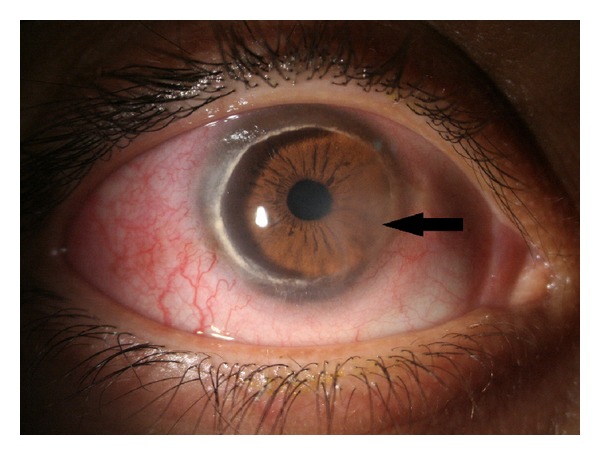
Photograph of the right eye showing conjunctival injection and circumferential peripheral gutter with opacification, lipid deposition, and vascularization. Mild edema also noted in the inferior-nasal portion of the cornea (solid black arrow).

**Figure 2:**
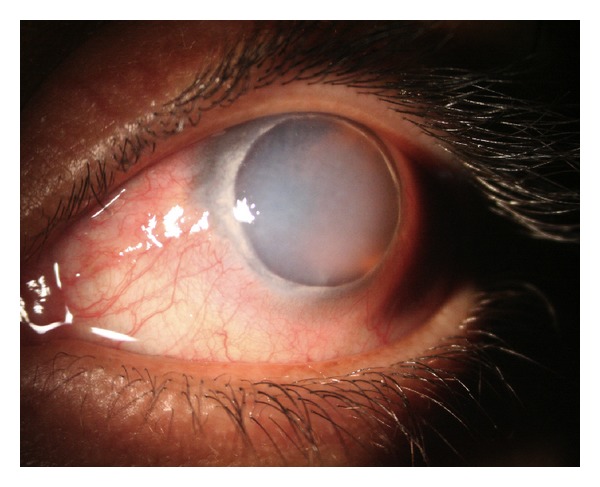
Photograph of the left eye demonstrating similar presentation to the right but with diffuse edema.

**Figure 3:**
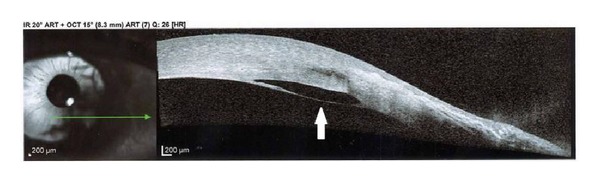
SPECTRALIS SD-OCT photograph of right eye showing partial detachment of Descemet's membrane (solid white arrow), cyst formation, and peripheral thinning.

**Figure 4:**
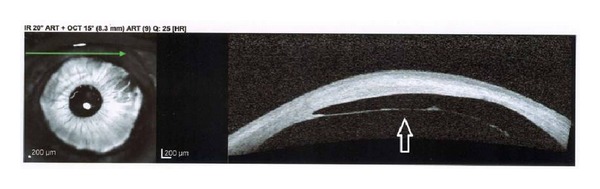
SPECTRALIS SD-OCT photograph of left eye demonstrating complete detachment of Descemet's membrane (white arrow outline).

Slit-lamp examination demonstrated conjunctival injection and circumferential peripheral thinning with intact overlying epithelium in both eyes (Figures 1 and 2). In her right eye, a vascular pannus traversed the area of stromal thinning with lipid deposition at its leading edge. Mild edema was present in the inferior-nasal portion of the right cornea (Figure 1). The posterior aspect of the cornea showed an intralamellar fluid cyst in the inferior nasal region anterior to Descemet's membrane. Fundus exam in her right eye was unremarkable. The left eye had significant corneal edema throughout all quadrants (Figure 2). Imaging with the SPECTRALIS SD-OCT with Anterior Segment Module (Heidelberg Engineering, Heidelberg, Germany) found partial Descemet's detachment in the right eye and complete detachment in the left eye (Figures 3 and 4). Keratometric readings were 44.7 @ 144°, 40.1 @ 54° in her right eye and 50.1 @ 77°, 41.6 @ 167° in her left eye. Ocular pressure was ten mmHg bilaterally, and Schirmer's test with and without anesthetic drops was within normal limits.

The patient's left eye was treated with an intracameral injection of air using a 30 gauge needle to maximally fill the anterior chamber. The procedure was performed under local anesthetic while in the supine position. An examination conducted eight hours postoperatively showed significant corneal improvement with visualization of the iris. The patient was placed on topical betamethasone and chloramphenicol for one month following the procedure. Her cornea was clear at one week with her visual acuity returning to 20/40 in both eyes. During the three years following this treatment, the patient had three additional occurrences of bilateral sectorial edema that waxed and waned but had no overt signs of a complete detachment of Descemet's membrane. These episodes each resolved spontaneously using topical NaCl 5% ointment and drops. An additional matter that merits mention is related to the sustained absence of observed intraocular inflammation during initial presentation, as well as throughout the entire follow-up period.

## 3. Discussion

Significant findings in this patient include bilateral complete circumferential thinning and ectasia of the cornea, total and partial detachment of Descemet's membrane, recurrent corneal edema, and a history of juvenile idiopathic arthritis. It is important to consider alternate explanations for the patient's presentation since TMD is closely associated with a family of peripheral corneal thinning disorders both noninflammatory and inflammatory in nature. A listing of plausible conditions within our differential diagnosis included keratoconus, pellucid marginal degeneration, Mooren's ulcer, Fuchs superficial marginal keratitis, and peripheral ulcerative keratitis (PUK).

Noninflammatory conditions such as keratoconus and pellucid marginal degeneration are improbable due to the patient's striking gutter formation with lipid keratopathy and a lack of either central [5] or isolated inferior ectasia [6]. Mooren's ulcer is not likely because it is characterized by severe pain, corneal thinning with an “overhanging” edge, and disruption of the epithelium in the absence of a systemic disease [1, 4, 7].

**Figure 5:**
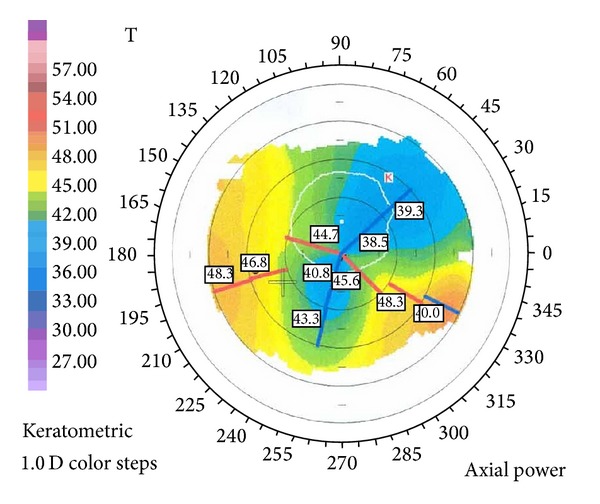
Orbscan photograph of right eye showing irregular astigmatism.

**Figure 6:**
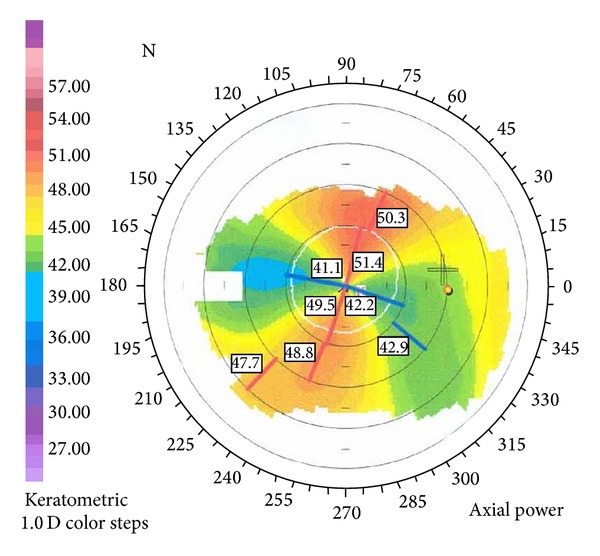
Orbscan photograph of left eye demonstrating irregular astigmatism and vertical steepening.

Given the patient's underlying systemic disease process, the possible diagnosis of peripheral ulcerative keratitis is important to examine. There have actually been very few case reports of PUK in patients with juvenile idiopathic arthritis [8–10]. Peripheral ulcerative keratitis has a diverse group of symptoms but key differences that distinguish it from this case include pain, tearing, photophobia, crescent-shaped ulcer, and, most importantly, destruction of the epithelium [11–13]. We believe that bilateral circumferential corneal thinning, opacification, lipid deposition, and vascularization paint a persuasive clinical picture for TMD. The results of the Orbscan (Orbtek, Inc., Salt Lake City, UT) demonstrating a high degree of irregular astigmatism strengthen this diagnosis (Figures 5 and 6). The concurrent diagnosis of JIA is unique but may actually be exacerbating the patient's eye disease. Of note, JIA as a distinct entity may engender several adverse ophthalmologic conditions, including increased propensity for keratoconjunctivitis sicca [14] and development of uveitis, with an estimated incidence affecting 10–20% of patients [15, 16]. Keratoconjunctivitis sicca may induce conjunctival injection and contribute to substandard visual acuity, each of which was illustrated during initial examination of our patient. Potential sequelae resulting from uveitis include various structural complications (i.e., epiretinal formation and band keratopathy) and loss of vision [4].

This brings up an interesting trend in the literature that is worth being explored. The argument for a second type of TMD has been made previously and is characterized by a younger age of initial presentation and recurrent attacks of episcleritis and scleritis [3]. While obvious signs of inflammation have been a defining feature for this variant, subclinical signs of inflammation may play an important role as well. In their respective patients, Ferrari et al. [17] and later Ceresara et al. [18] were able to demonstrate inflammation at the cellular level using laser scanning in vivo confocal microscopy even when no outward manifestations of inflammation were found. Evidence of inflammatory cell infiltration in the stroma, activated keratocytes, and abnormal structure of the subbasal nerve plexus all supported this idea. Both papers also referred to the similarity of these findings with systemic inflammatory diseases like Sjögren syndrome and rheumatoid arthritis. Cases of TMD in the setting of systemic inflammatory disease are rare but have been reported in rheumatoid arthritis [19, 20] and erythema elevatum diutinum [21]. Considered collectively, it is the hypothesis of these authors that inflammatory changes contribute to the pathological process of TMD and cases of systemic inflammatory disease can accelerate the process. It must be mentioned that our immunologic workup was not entirely comprehensive, and further analysis to thoroughly exclude conditions such as endothelial dystrophy and peripheral ulcerative keratitis still requires attention.

The other important element of this patient's presentation is the development and progression of cystic changes and detachment of Descemet's membrane in each eye. A wide range of changes in the posterior aspect of the cornea have been documented but to our knowledge have never involved complete detachment of Descemet's membrane in the setting of TMD. These changes include thickening, thinning, breaks, bands, and holes in Descemet's membrane [5, 18, 20]. Posterior polymorphous dystrophy [20, 22] and corneal cyst [23] formation have both consequently been described in case reports with TMD. If we then include our patient, we see a continuum starting with small vesicular changes, moving to cyst formation, and finally partial and complete detachment of Descemet's membrane. The waxing and waning course of our patient's edema may indicate some correlation in the degree to which her JIA is being controlled. With regard to the occurrence of Descemet's membrane detachment, we hypothesize that a significant dissimilarity between the degree of induced anterior corneal-curvature variation and posterior curvature variation occurred, resulting in a discrepancy between respective shearing forces generated within each surface. This asymmetric vectorial configuration may have resulted in endothelial separation and concurrent detachment of Descemet's membrane. Apart from this conjectured proposal, we are uncertain of the true underlying mechanism regarding spontaneous Descemet's detachments within this patient.

To the best of our knowledge, this is the first reported case of Terrien's marginal degeneration associated with either juvenile idiopathic arthritis or Descemet's membrane detachment. We believe this presentation gives insight into both the pathogenesis and progression of this unique disorder.
